# Quantitative assessment of enlarged perivascular spaces via deep-learning in community-based older adults reveals independent associations with vascular neuropathologies, vascular risk factors and cognition

**DOI:** 10.1093/braincomms/fcae252

**Published:** 2024-07-30

**Authors:** Carles Javierre-Petit, Marinos Kontzialis, Sue E Leurgans, David A Bennett, Julie A Schneider, Konstantinos Arfanakis

**Affiliations:** Department of Biomedical Engineering, Illinois Institute of Technology, Chicago, IL 60616, USA; Department of Diagnostic Radiology and Nuclear Medicine, Rush University Medical Center, Chicago, IL 60612, USA; Rush Alzheimer’s Disease Center, Rush University Medical Center, Chicago, IL 60612, USA; Department of Neurological Sciences, Rush University Medical Center, Chicago, IL 60612, USA; Rush Alzheimer’s Disease Center, Rush University Medical Center, Chicago, IL 60612, USA; Department of Neurological Sciences, Rush University Medical Center, Chicago, IL 60612, USA; Rush Alzheimer’s Disease Center, Rush University Medical Center, Chicago, IL 60612, USA; Department of Neurological Sciences, Rush University Medical Center, Chicago, IL 60612, USA; Department of Pathology, Rush University Medical Center, Chicago, IL 60612, USA; Department of Biomedical Engineering, Illinois Institute of Technology, Chicago, IL 60616, USA; Department of Diagnostic Radiology and Nuclear Medicine, Rush University Medical Center, Chicago, IL 60612, USA; Rush Alzheimer’s Disease Center, Rush University Medical Center, Chicago, IL 60612, USA

**Keywords:** perivascular spaces, pathology, MRI, deep-learning, cognition

## Abstract

Enlarged perivascular spaces (EPVS) are common in older adults, but their neuropathologic correlates are unclear mainly because most work to date has relied on visual rating scales and/or clinical cohorts. The present study first developed a deep-learning model for automatic segmentation, localization and quantification of EPVS in *ex vivo* brain MRI, and then used this model to investigate the neuropathologic, clinical and cognitive correlates of EPVS in 817 community-based older adults that underwent autopsy. The new method exhibited high sensitivity in detecting EPVS as small as 3 mm^3^, good segmentation accuracy and consistency. Most EPVS were located in the frontal lobe, but the highest density was observed in the basal ganglia. EPVS in the cerebrum and specifically in the frontal lobe were associated with infarcts independent of other neuropathologies, while temporal and occipital EPVS were associated with cerebral amyloid angiopathy. EPVS in most brain lobes were also associated with diabetes mellitus independently of neuropathologies, while basal ganglia EPVS were independently associated with hypertension, supporting the notion of independent pathways from diabetes and hypertension to EPVS. Finally, EPVS were associated with lower cognitive performance independently of neuropathologies and clinical variables, suggesting that EPVS represent additional abnormalities contributing to lower cognition.

## Teaser

Javierre-Petit *et al*. assessed enlarged perivascular spaces (EPVS) via deep-learning in 817 community-based older adults who underwent autopsy and revealed the vascular pathologies and risk factors linked to EPVS, as well as indicated that tissue damage associated with EPVS has independent contributions to lower levels of cognition.

## Graphical Abstract

**Graphical Abstract:**
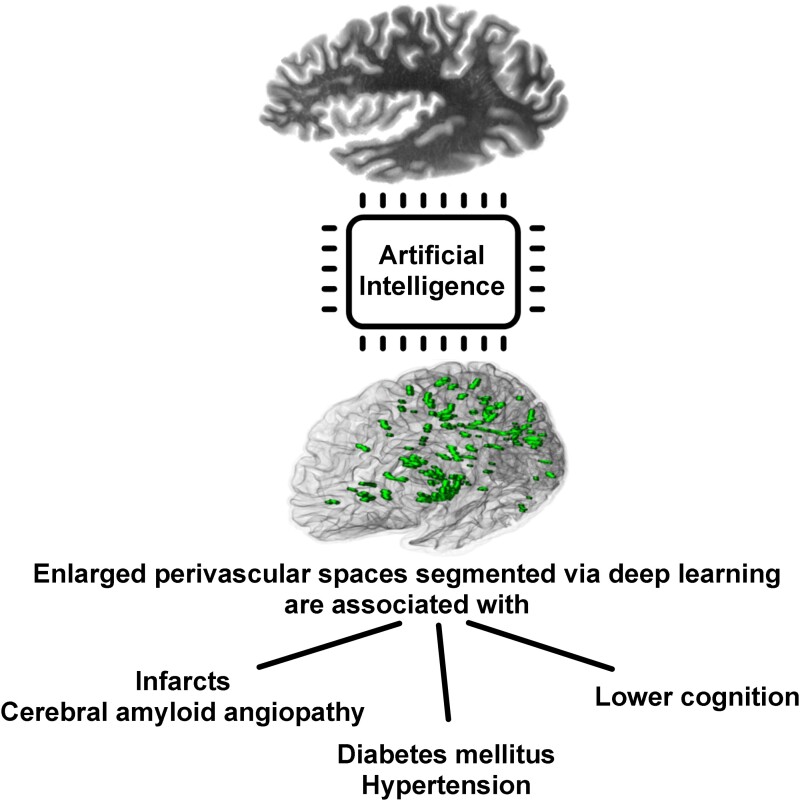

## Introduction

Perivascular spaces (PVS) are fluid-filled spaces that surround cerebral blood vessels and facilitate interstitial fluid exchange and clearance of waste products as part of the brain’s glymphatic system.^1-4^ Enlarged perivascular spaces (EPVS) are common in older adults and can be detected in MRI as hyperintense linear features in T_2_-weighted images, or as hypointense features in T_1_-weighted and T_2_-weighted fluid-attenuated inversion recovery images.^5-7^ Risk factors for EPVS include advancing age,^8^ hypertension^9^ and diabetes.^10^ EPVS have been associated with an increased risk of stroke,^11^ MRI markers of small vessel disease such as white matter hyperintensities and microbleeds,^12,13^ as well as cognitive decline,^14^ mild cognitive impairment (MCI) and dementia.^15,16^ The proposed mechanisms that may lead to EPVS include protein aggregation,^17^ arterial stiffening and perivascular inflammation.^18,19^ However, the neuropathologic correlates of EPVS in community-based older adults are not well understood.

An association of EPVS with cerebral amyloid angiopathy (CAA) has been shown in MRI-pathology investigations conducted in patients mainly suffering from CAA.^20,21^ In the largest MRI-pathology investigation of EPVS conducted to date, we showed that EPVS burden in community-based older adults is associated with pathologically diagnosed infarcts.^10^ Also, studies combining MRI and fluid-based or PET-based markers, but not pathology, have shown that EPVS in the central semiovale are associated with annual change of plasma P-tau_181_ and tau positivity in cognitively normal older adults,^22,23^ and with amyloid beta (Aβ) positivity in patients with cognitive impairment.^24^ The above discrepancies in findings may be due to a number of differences across studies including differences in the type of sample (clinic-based or community-based), in methodology for MRI data acquisition and neuropathological examination, and in the approach for handling mixed pathologies commonly co-occurring in the older adult brain. Another important factor that may have contributed to the inconsistent findings across studies is the use of different visual rating scales for assessing EPVS. Full quantification of EPVS was not used in the above-mentioned MRI-pathology investigations because manual segmentation of EPVS is time-consuming, and the fully automated algorithms published recently may have not been available or may have not been applicable to the MRI data collected in those studies.^6,7,25^ Quantitative assessment of EPVS can address important limitations of visual rating scales such as detection bias and floor/ceiling effects, greatly improving the accuracy and consistency of findings across investigations.

The purpose of this work was 2-fold: (i) to develop a deep-learning model for automatic segmentation and quantification of EPVS in *ex vivo* brain MRI data, and (ii) to use this new method to investigate the neuropathologic, clinical and cognitive correlates of EPVS in a large number of community-based older adults with available *ex vivo* brain MRI and detailed pathology, clinical and cognitive data. This study used *ex vivo* instead of *in vivo* MRI as *ex vivo* MRI captures brain characteristics at the same condition of the brain as neuropathologic examination, and also reduces the inherent bias of *in vivo* MRI against frail individuals. First, a deep-learning model for automatic segmentation and quantification of EPVS in *ex vivo* brain MRI data was developed as available tools are not applicable to *ex vivo* MRI. Next, the neuropathologic, clinical and cognitive correlates of EPVS were investigated in a large number of community-based older adults by combining fully quantitative *ex vivo* assessments of EPVS in the whole brain as well as in individual lobes with detailed neuropathologic, clinical and cognitive data. More specifically, detailed pathologic examination assessed gross and microscopic infarcts, CAA, atherosclerosis, arteriolosclerosis, Αβ, neurofibrillary tangles, Lewy bodies and Limbic-predominant age-related TDP-43 encephalopathy-neuropathological change (LATE-NC). Cognition was assessed longitudinally, and the independent association of EPVS with level of cognition and cognitive decline above and beyond the effects of neuropathologies, risk factors and demographics was tested.

## Materials and methods

### Participants

This study included older adults from four longitudinal, epidemiologic, clinical-pathologic cohort studies of aging, the Rush Memory and Aging Project (MAP),^26^ the Religious Orders Study (ROS), the Minority Aging Research Study (MARS)^27^ and the African American Clinical Core of the Rush Alzheimer’s Disease Research Center.^28^ All four studies were approved by an Institutional Review Board of Rush University Medical Center. Participants provided written informed consent according to the Declaration of Helsinki and signed an anatomical gift act. All participants underwent annual uniform structured clinical evaluations, including cognitive function testing, medical history and neurologic examination,^29^ and after death underwent autopsy, *ex vivo* brain MRI, and detailed neuropathologic examination. At the time of these analyses, 5067 participants of the parent projects had completed the baseline evaluation. Of these, 620 died and 123 withdrew from the studies before the *ex vivo* MRI sub-study began. Of the remaining 4324 persons, 1593 died, 1290 were autopsied and 1039 had *ex vivo* MRI and pathology data. The first 831 consecutive participants with *ex vivo* MRI and pathology data were considered in this study, of whom 14 were excluded due to widespread brain abnormalities (e.g. large infarcts, tumours) preventing visualization of EPVS. The remaining 817 participants were included in analyses.

### Cognitive function testing and clinical diagnosis

Cognitive function was assessed annually with a battery of 17 cognitive tests targeting five cognitive domains: episodic memory, semantic memory, working memory, perceptual speed and visuospatial ability.^26^ Composite scores for each domain as well as for global cognition were constructed by averaging *Z*-scores from individual tests within each cognitive domain, as well as over all tests. Clinical diagnosis of dementia followed the criteria established by the National Institute of Neurological and Communicative Disorders and Stroke and the Alzheimer’s Disease and Related Disorders Association.^30^ Participants who did not meet the criteria for dementia despite having cognitive impairment were classified as having MCI,^31,32^ whereas participants with neither dementia nor MCI were classified as having no cognitive impairment (NCI).^33^ At the time of death, a neurologist reviewed all available clinical data and rendered a summary final diagnostic opinion, blinded to all post-mortem data.

### Brain hemisphere preparation

At autopsy, the brain was removed and the cerebral hemisphere with more visible pathology was selected for *ex vivo* MRI and pathologic examination, while the contralateral hemisphere was frozen and stored.^34^ The selected hemisphere was immersed in phosphate-buffered 4% formaldehyde solution and refrigerated at 4°C within 30 min after removal from the skull. Prior to *ex vivo* MRI, the refrigerated hemisphere was positioned in a container filled with 4% formaldehyde solution with its medial aspect facing the bottom of the container and allowed to return to room temperature. Gross examination and histopathologic diagnostic examination of the hemisphere were performed within 2 weeks after *ex vivo* MRI.^35^

### *Ex vivo* MRI data acquisition

*Ex vivo* MRI data were collected ∼30 days post-mortem using 3 Tesla MRI scanners. Four MRI scanners were used in this work because of scanner upgrades during the more than 12 years of data collection. Nevertheless, a two-dimensional multi-echo spin-echo sequence with the same voxel-size (0.6 mm × 0.6 mm × 1.5 mm) and similar values for all other parameters was used on all scanners (see detailed protocols of Arfanakis *et al*.^34^), and only T_2_-weighted images collected at echo times between 50 and 58 ms were used in the analysis to maintain consistency in image contrast across scanners.

### Neuropathologic evaluation

Following *ex vivo* MRI, each hemisphere underwent detailed neuropathologic examination by a board-certified neuropathologist blinded to all imaging and clinical data. First, coronal slabs (1 cm thick) of each hemisphere were macroscopically evaluated. Next, tissue blocks were selected, embedded in paraffin, sectioned at 6 µm thickness and stained with haematoxylin and eosin for microscopic evaluation. Gross infarcts of any age were rated as present (1) or absent (0).^36^ Microscopic infarcts of any age were identified from a minimum of nine brain regions (including midfrontal, middle temporal, entorhinal, anterior cingulate, inferior parietal cortices, hippocampus, thalamus, basal ganglia and midbrain) and were also rated as present (1) or absent (0).^37^ CAA was assessed in four regions (midfrontal, middle temporal, angular and calcarine cortices) and was scored as none (0), mild (1), moderate (2) or severe (3).^38^ Arteriolosclerosis was evaluated in sections of the anterior basal ganglia and was rated as none (0), mild (1), moderate (2) or severe (3).^39^ Atherosclerosis was evaluated at the circle of Willis and was rated as none (0), mild (1), moderate (2) or severe (3).^39^ Aβ and neurofibrillary tangles were identified by molecularly specific immunohistochemistry (three monoclonal antibodies against Aβ, 4G8, 6F/3D, 10D5; antibodies to abnormally phosphorylated Tau protein, AT8) in eight brain regions (hippocampus, entorhinal, inferior temporal, angular gyrus, calcarine, anterior cingulate, superior frontal and midfrontal cortices) and composite measures of Aβ burden and tangle density were computed as previously described.^40^ A pathologic diagnosis of AD was also made using the National Institute on Aging-Alzheimer’s Association (NIA-AA) criteria.^41^ Lewy bodies were assessed in seven brain regions (entorhinal, anterior cingulate, midfrontal, superior or middle temporal, and inferior parietal cortices, substantia nigra and amygdala) and were rated as present (1) or absent (0).^42^ LATE-NC was graded into four stages: no TDP-43 inclusions (Stage 0); TDP-43 inclusions in amygdala only (Stage 1); TDP-43 inclusions in amygdala and entorhinal cortex or hippocampus (Stage 2); TDP-43 inclusions in amygdala, entorhinal cortex or hippocampus and neocortex (Stage 3).^43^

### Data pre-processing and feature extraction

For each participant, *ex vivo* T_2_-weighted MRI data were N4 bias-field-corrected^44^ and intensity-normalized by means of robust *Z*-scores (using the 2.5–97.5 percentile range). Masks of the brain hemisphere, white matter, frontal lobe, parietal lobe, occipital lobe, temporal lobe and basal ganglia were produced using in-house algorithms. A set of uncorrelated EPVS-enhanced image volumes was generated using the following methodology. First, pre-processed MR images were denoised using a 4D block-matching technique.^45^ Next, multi-scale features were generated using steerable filters (SF), Frangi filters (FF)^46^ and optimally oriented flux (OOF).^47^ Specifically, SF used 9 orientations for first- and second-order Gaussian derivatives and also used the Gaussian response thereby generating a total of 19 features per scale (i.e. per width of the Gaussian), FF and OOF each generated five features per scale, and three scales were selected for SF, 12 for FF and 12 for OOF, for a total of 177 multi-scale features.^48^ The same process was also applied on a white top-hat-transformed version of the pre-processed MR images generating 177 additional features, for a total of 354 features per participant. Finally, principal component analysis (PCA) was used on the 354 features, and the first 12 components accounting for 80% of the variance were chosen and are referred to as PCA features in the following.

### Deep-learning model

**Figure 1:**
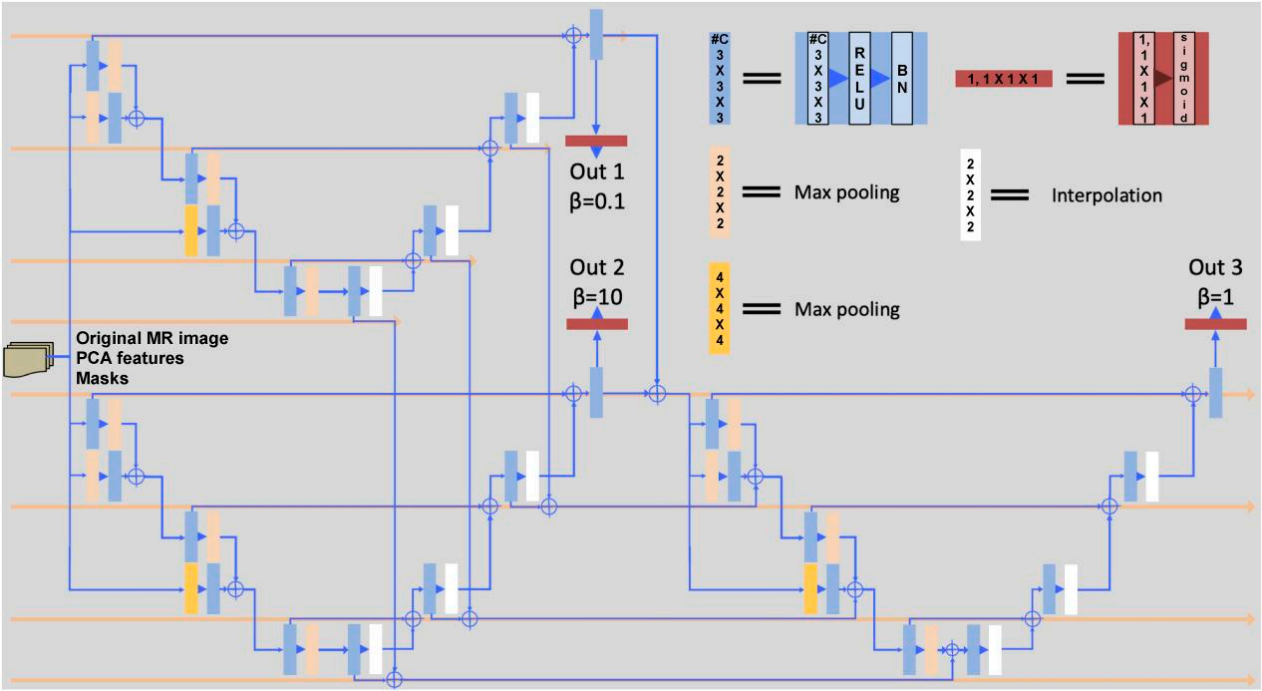
**Architecture of the deep-learning model.** Architecture of the deep-learning model for EPVS segmentation (see detailed description in Sections titled ‘Deep-learning model’ and ‘Model training’). PCA, principal component analysis; ReLU, rectified linear unit; BN, batch normalization.

A deep-learning convolutional neural network (CNN) was developed and optimized to automatically segment EPVS in *ex vivo* MRI data. The model was fully convolutional and consisted of three U-Nets organized in a two-stage layout (Fig. 1). The first stage consisted of two U-Nets arranged in parallel at the input, and the second stage included a U-Net that merged feature maps from the first stage. Skip connections between U-Nets were present at every level of the encoder–decoder stack and all skip connections used a concatenation operation (represented by the + sign in Fig. 1). The input data to the first stage were 19 three-dimensional image volumes consisting of the pre-processed MR images, the 12 EPVS-enhanced PCA features, the white matter mask, and the five lobar masks. The CNN model consisted of ∼5 million trainable parameters.

The three U-Nets were identical to each other and were based on the M2EDN architecture with modifications in the optimization process.^49^ Each U-Net consisted of multiple convolutional layers, pooling layers and up-sampling layers. In the encoder sub-network, each convolutional block (shown as blue rectangles in Fig. 1) consisted of 64 channels with 3 × 3 × 3 convolutional kernels with a stride of 1 and zero padding, followed by a rectified linear unit (ReLU) activation function and a batch normalization (BN) layer.^50,51^ Max pooling layers (shown as orange rectangles in Fig. 1) were 2 × 2 × 2 with a stride of 2 (except for one 4 × 4 × 4 with a stride of 4 for direct input into the second level of the encoder; shown as bright orange rectangles in Fig. 1). Network inputs were down-sampled three times in the encoder sub-network. Symmetric to the encoder sub-network, the decoder sub-network consisted of operations arranged at three increasing resolution levels. Convolutional blocks were the same as those described above. Up-sampling was executed by interpolation layers (shown as white rectangles in Fig. 1) using 2 × 2 × 2 kernels with a stride of 2 and nearest neighbour interpolation. At each resolution level, a skip connection was included to fuse the up-sampled feature maps with the same level feature maps obtained from the previous encoder sub-network. Finally, output layers (shown as red rectangles in Fig. 1) were single channel 1 × 1 × 1 convolutional kernels with a stride of 1 followed by a sigmoid activation function (bounded [0,1] output) to calculate voxel-wise EPVS probability maps from high-dimensional feature maps. Multi-scale features were also implemented in the encoder sub-network. The first two decreasing resolution levels implemented convolution followed by a max pooling module and simultaneously a module with max pooling followed by convolution. This allowed for parallel quantification of features using different scales at each resolution level, which were then fused and used as input to the subsequent resolution level.

### Model training

*Ex vivo* MRI data from 10 participants with varying EPVS severity and manually segmented EPVS were used to train the CNN model. Data from nine participants were used for training and the remaining data were used for validation. The training process consisted of 1200 epochs, with 40 batches per epoch and 8 samples per batch. In each training batch, samples were randomly generated on the fly from the training dataset and then fed into the model. Model validation used a fixed set of overlapping samples from the validation participant to ensure consistency when tracking model performance. At the end of each epoch, the validation loss was recorded, and the model coefficients were stored. This training process was repeated 10 times using leave-one-out cross-validation (LOOCV).

The model was trained using a F(β) loss function:


$$F(\beta )=1-\frac{(1+\beta )\sum_{i}\;y_{i}{\hat{y}}_{i}+\varepsilon }{\beta \sum_{i}\;y_{i}+\sum_{i}\;{\hat{y}}_{i}+\varepsilon }$$

where $y_{i}$ is the manually segmented EPVS label for the *i*th voxel taking values of 0 or 1, ${\hat{y}}_{i}$ is the predicted label probability for the *i*th voxel taking real values in the range [0,1], *β* controls the effect of precision and recall in minimizing the loss and ε is a small scalar (10^−3^ here) to maintain numerical stability. Different *β* values were used in each of the three U-Nets. The two parallel U-Nets in the first stage used *β* parameters of 10 and 0.1 to encourage learning of features with high recall and high precision, respectively. The third U-Net in the second stage used β=1 to learn features that balanced precision and recall by combining the learned features from the two previous U-Nets.

Multiple regularization strategies were implemented to minimize overfitting. Each convolutional layer used BN and DropConnect.^51,52^ An L2 regularization (weight decay) on convolutional filters was used. During training, a patch-based approach was also implemented as follows. For each sample in a mini-batch, a participant within the training set was randomly selected, a voxel within the participant’s white matter mask was chosen, and a 64 × 64 × 24 patch centred at the selected white matter voxel was extracted. Allowing every white matter voxel and its surrounding patch to be sampled essentially generated as many unique training samples as the number of white matter voxels, dramatically increasing the number of training samples and reducing overfitting. Finally, early stopping was implemented by saving model weights at every epoch and selecting the set of weights that maximized the Dice similarity coefficient (DSC) (after 0.5 thresholding of the output probabilities) on the entire validation image volume. Models were trained using a stochastic gradient descent optimizer with a learning rate λ=0.001 using Nesterov momentum with m=0.9.^53^ The kernel regularization parameter was set to $l_{2}=0.001$, and the DropConnect probability was set to $P_{\mathrm{drop}}=0.3$.

### Segmentation model ensemble

To protect against overfitting, an ensemble of 10 models was trained from the LOOCV process described above. The 10 EPVS probability maps generated from the individual models were averaged and appropriately thresholded to generate the final EPVS segmentation output for the ensemble. The optimal threshold was determined as follows. First, a new dataset was constructed by randomly erasing selected brain lobes (using the lobar masks) from each participant’s original data. Then, an ensemble was trained using LOOCV over this newly constructed dataset. Finally, optimal thresholding was found by evaluating the segmentation performance of the trained ensemble over the erased brain lobes (i.e. holdout data). This process was repeated five times, and the final threshold was the average of the optimal thresholds across the five trained ensembles. The final EPVS probability threshold value was 0.2.

### Ensemble performance evaluation

**Figure 2:**
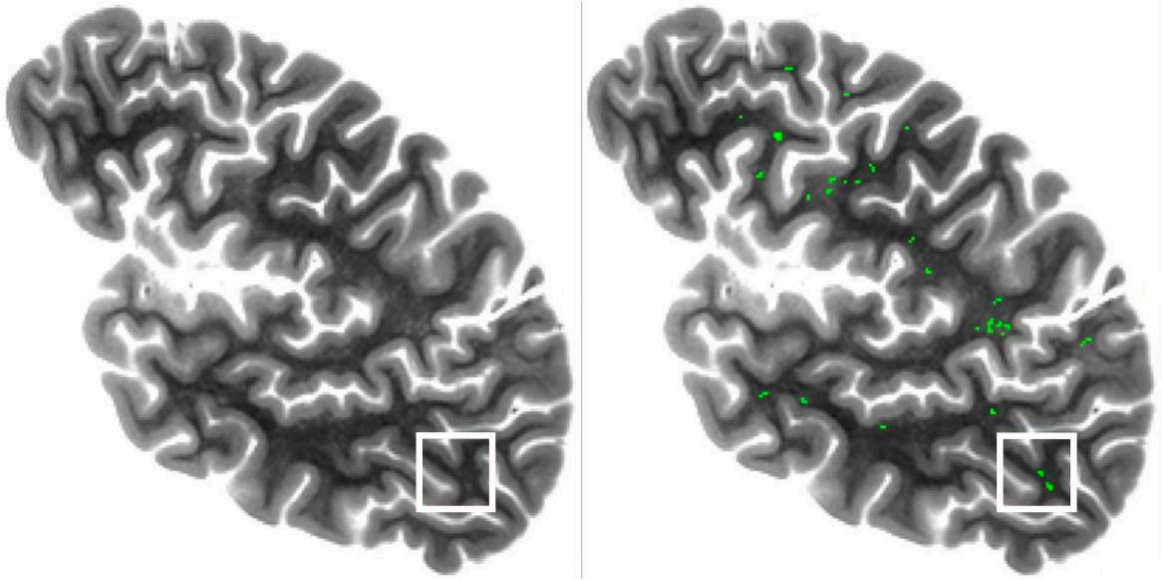
**Examples of materials used for ensemble performance evaluation.** Example of a ROI (white square) (sized 25 × 25 voxels in-plane and nine voxels through-plane; 25 voxels is equivalent to 15 mm) generated for ensemble performance evaluation and examples of manually segmented EPVS (green).

Evaluation of ensemble performance involved a board-certified neuroradiologist as well as an experienced observer. First, to facilitate the evaluation, 100 regions of interest [ROIs (sized 25 × 25 × 9 voxels)] that included hyperintense white matter structures with intensities similar to those of the manually segmented EPVS of the training data were randomly generated in 100 unique participants (one ROI per participant) using an automated algorithm (Fig. 2). To promote a balanced ROI distribution in the brain, 20 ROIs were located within each lobe and the basal ganglia (for a total of 20 × 5 = 100 ROIs). Ensemble sensitivity was evaluated as follows. A board-certified neuroradiologist reviewed all ROIs, identified EPVS, and selected multiple voxels belonging to each EPVS (rough segmentation of each EPVS). Next, an experienced observer assisted with detailed manual segmentation of the neuroradiologist-identified EPVS. Both the neuroradiologist and experienced observer were blinded to the output of the ensemble. If at least one voxel of a manually segmented EPVS was also segmented by the ensemble, then that EPVS was marked as successfully detected by the ensemble. Sensitivity was calculated as the proportion of the neuroradiologist-identified EPVS that were detected by the ensemble across all ROIs. Ensemble segmentation accuracy was quantified using the DSC between the neuroradiologist-identified, manually segmented EPVS and the output of the segmentation model ensemble considering all ROIs. Ensemble segmentation consistency was measured using Pearson’s correlation coefficient between the number of manually segmented voxels and the number of automatically segmented voxels in each ROI.

### Application of the segmentation model ensemble

The developed ensemble was used to automatically segment EPVS in all 817 participants. The number of EPVS in the cerebrum as well as in individual lobes, and the basal ganglia was measured for each participant. To mitigate skewness, EPVS counts in individual lobes and the basal ganglia were used as is up to the median value (50th percentile) and higher counts were binned into four levels as follows: the range of EPVS counts in the 50th–75th percentile was divided into three equal intervals defining three levels, and EPVS counts higher than the value at the 75th percentile were binned into one final level. The same process was used to mitigate the skewness of EPVS counts in the cerebrum, with the addition that counts from the 0th to 50th percentile were also binned into four levels.

### Statistical analysis

Pearson and Spearman correlations were used to assess relationships between neuropathologies and between neuropathologies and clinical variables. Ordinal logistic regression was used to investigate the association of the number of EPVS in the cerebrum (dependent variable) with each of the neuropathologies (i.e. the presence of gross and microscopic infarcts, the severity of CAA, arteriolosclerosis and atherosclerosis, the composite measures of Aβ burden and tangle density, the presence of Lewy bodies and LATE-NC stage) and clinical variables (history of heart disease, hypertension, diabetes mellitus, smoking and presence of at least one copy of the *APOE* ε4 allele), controlling for demographics (age, sex and years of education), volume of the cerebral hemisphere, scanner and post-mortem intervals to fixation and to imaging. Next, all the variables that reached *P* < 0.20 in the first step were included together in a single ordinal logistic regression model where the number of EPVS in the cerebrum was again the dependent variable. The same two-step process was repeated for the number of frontal, parietal, temporal, occipital and basal ganglia EPVS as dependent variables (replacing in these models the volume of the cerebral hemisphere with the volume of the corresponding brain region). After applying Bonferroni correction to control for testing six ordinal logistic regression models (i.e. one for the number of EPVS in the cerebrum and one for the number of EPVS in each lobe and basal ganglia), associations were considered significant at *P* < 0.0083 (i.e. 0.05 divided by 6).

Linear mixed-effects models were used to investigate the independent association of the number of EPVS in the cerebrum with the level of cognition at the time of death and the rate of cognitive decline above and beyond what was explained by neuropathologies, clinical variables and demographics. Models included a composite cognitive score (for global cognition or each of the five cognitive domains) as the longitudinal dependent variable, the number of EPVS in the cerebrum as well as its interaction with the time before death as the independent variables, and were controlled for all the neuropathologies, clinical variables, demographics and volume of the cerebral hemisphere, as well as the interaction of each of these variables with the time before death. Associations of the number of EPVS in the cerebrum with cognition at death and the rate of cognitive decline were Bonferroni-corrected and considered significant at *P* < 0.0083 (i.e. 0.05 divided by 6 cognitive domains).

## Results

### Ensemble performance evaluation

**Figure 3:**
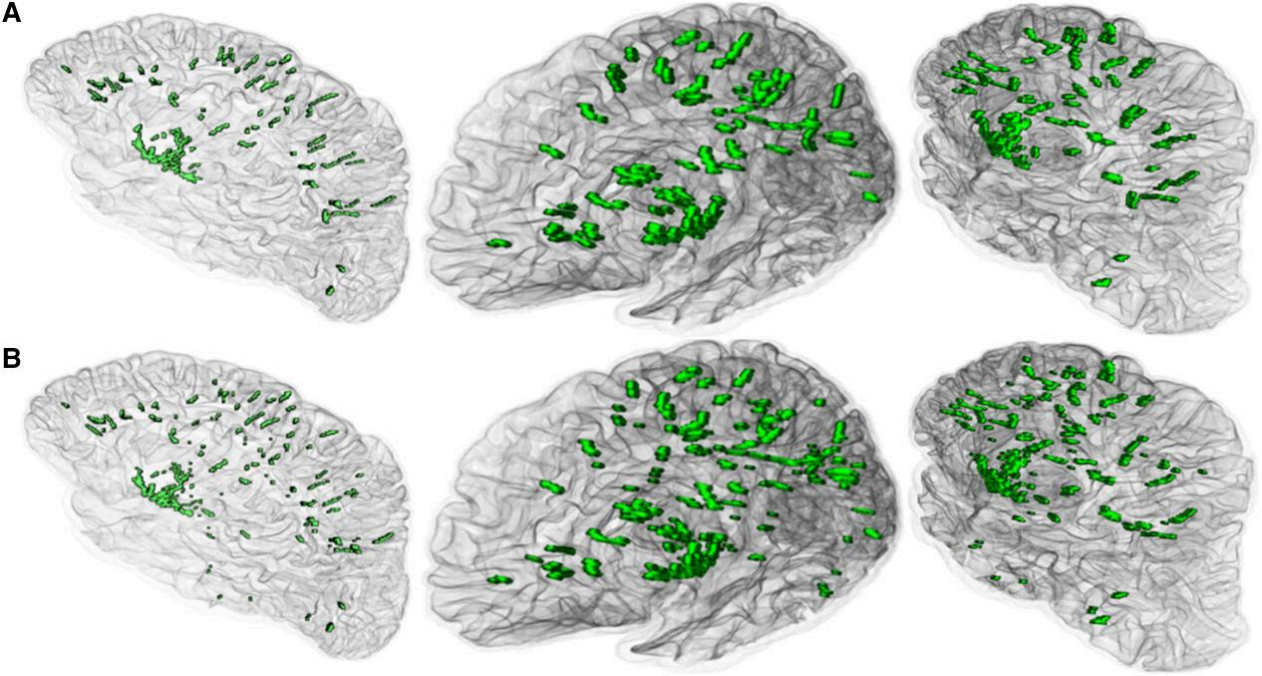
**Examples of manually and automatically segmented EPVS.** Examples of (**A**) manually and (**B**) automatically segmented EPVS presented in green colour for three rotations of a cerebral hemisphere from one of the participants.

When evaluating ensemble sensitivity, the neuroradiologist identified 351 EPVS across the 100 ROIs, and the ensemble correctly identified 237 EPVS, resulting in an overall detection rate of 68%. When considering the size of the EPVS, the detection rate was 80% for EPVS larger than 3 mm^3^ and higher than 90% for EPVS larger than 12 mm^3^. Therefore, the ensemble showed high sensitivity in detecting EPVS as small as 3 mm^3^. When evaluating ensemble segmentation accuracy, the experienced observer manually segmented 4392 voxels across the 100 ROIs, and the ensemble correctly segmented 2992 voxels, did not segment the remaining 1400 voxels, and incorrectly segmented 1634 voxels. Therefore, our EPVS segmentation ensemble achieved a DSC of 0.66. Furthermore, ensemble segmentation consistency was strong with a Pearson’s correlation of *ρ* = 0.91 (*P* < 10^−10^) between the numbers of manually and automatically segmented voxels in each ROI. An example of manually and automatically segmented EPVS for one of the participants is shown in Fig. 3.

### Demographic, clinical, neuropathologic and EPVS characteristics

In these 817 community-based older adults, the mean age at death was 90 years, 70% were female, about a third had NCI, a quarter MCI, and nearly half had dementia, the mean Mini-mental state examination (MMSE) at the last clinical evaluation was 20.0, and the mean global cognition score was −1.0 [SD = 1.2 (Table 1)]. The median time between the last clinical evaluation and death was 0.6 years, and the median post-mortem interval to immersion of tissue in fixative solution was 7.3 h (Table 1). In terms of neuropathologic characteristics, 68% of participants had high or intermediate AD neuropathologic change, 40% had gross infarcts, 38% microscopic infarcts and 34% moderate or severe CAA (Table 2). Correlations between neuropathologies and between neuropathologies and clinical characteristics were low (the highest Spearman correlations were: 0.53 between Aβ burden and tangle density, 0.37 between arteriolosclerosis and atherosclerosis and 0.34 between Aβ burden and CAA severity; all other Spearman correlations were <0.3 in absolute value).

**Table 1:** Demographic and clinical characteristics of the participants

*N*	817
Age at death, years, mean (SD)	90.3 (6.6)
Male, *n* (%)	235 (29%)
Education, years, mean (SD)	15.7 (3.6)
Median time between last clinical evaluation and death, years	0.6
MMSE, mean (SD)	20.0 (9.5)
NCI, *n* (%)	256 (31%)
MCI, *n* (%)	188 (23%)
Dementia, *n* (%)	373 (46%)
Global cognition, mean (SD)	−1.0 (1.2)
Episodic memory, mean (SD)	−1.0 (1.4)
Semantic memory, mean (SD)	−1.3 (1.7)
Working memory, mean (SD)	−0.7 (1.1)
Perceptual speed, mean (SD)	−1.2 (1.2)
Visuospatial ability, mean (SD)	−0.6 (1.2)
Heart disease, *n* (%)	155 (19%)
Hypertension, *n* (%)	576 (71%)
Diabetes mellitus, *n* (%)	180 (22%)
Smoking, *n* (%)	258 (32%)
Systolic blood pressure, mm Hg, mean (SD)	126 (21)
Diastolic blood pressure, mm Hg, mean (SD)	71 (11)
At least one copy of the ε4 allele, *n* (%)	209 (26%)
Right hemisphere, *n* (%)	371 (45%)
Post-mortem interval to fixation, h, mean (SD)	9.8 (7.8)
Post-mortem interval to imaging, d, mean (SD)	37.4 (18.1)

**Table 2:** Neuropathologic characteristics of the participants

*N*	817
Gross infarcts, *n* (%)	327 (40%)
Microscopic infarcts, *n* (%)	313 (38%)
Cortical microscopic infarcts, *n* (%)	210 (26%)
Subcortical microscopic infarcts, *n* (%)	149 (18%)
CAA, *n* (%)	
Severe	92 (11%)
Moderate	190 (23%)
Mild	356 (44%)
Arteriolosclerosis, *n* (%)	
Severe	48 (6%)
Moderate	167 (20%)
Mild	317 (39%)
Atherosclerosis, *n* (%)	
Severe	44 (5%)
Moderate	160 (20%)
Mild	420 (51%)
AD-NC (NIA-AA), *n* (%)	
High or intermediate	557 (68%)
Lewy bodies, *n* (%)	238 (29%)
LATE-NC, *n* (%)	
Stage 3	213 (26%)
Stage 2	92 (11%)
Stage 1	144 (18%)

**Figure 4:**
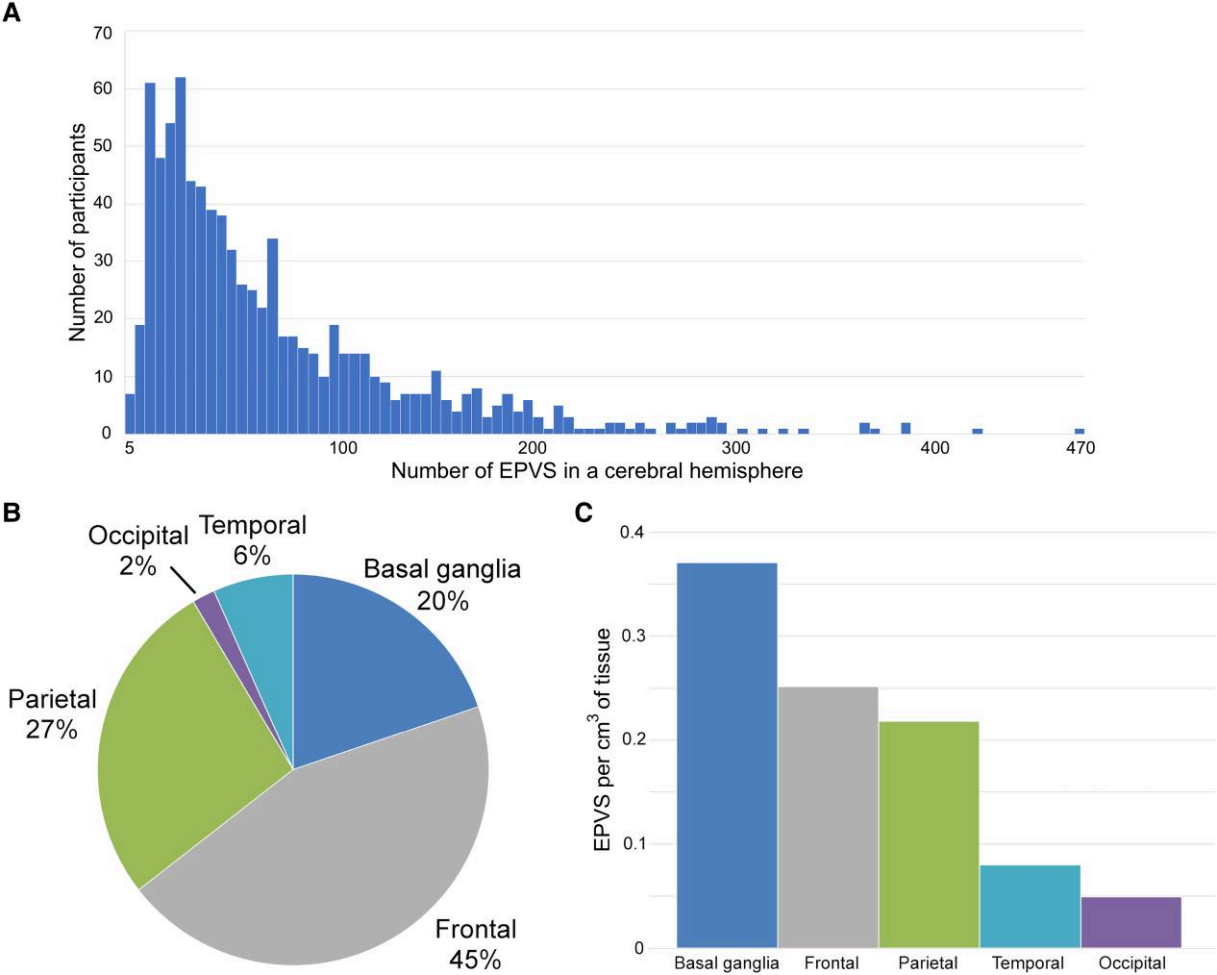
**Descriptive of automatically segmented EPVS**. (**A**) Histogram of the number of participants at different numbers of EPVS in the cerebrum (median number of EPVS per participant = 51; maximum number of EPVS in a participant = 469; total number of EPVS over all participants = 60 724; total number of participants = 817). (**B**) Piechart showing the percentage of all EPVS detected in different lobes and in the basal ganglia. (**C**) Density of EPVS (number of EPVS per cm^3^ of tissue) in different lobes and in the basal ganglia.

A total of 60 724 EPVS were automatically segmented over all participants. The median number of EPVS per participant was 51 and the maximum was 469 (Fig. 4A). Out of all the EPVS segmented, 45% were located in the frontal lobe, 27% in the parietal lobe, 2% in the occipital lobe, 6% in the temporal lobe and 20% in the basal ganglia (Fig. 4B). However, after normalizing the number of EPVS per lobe and in the basal ganglia with the average volume of the corresponding region, the resulting density of EPVS was highest in the basal ganglia (0.37 per cm^3^), followed by the frontal (0.25 per cm^3^), parietal (0.22 per cm^3^), temporal (0.08 per cm^3^) and occipital lobes (0.05 per cm^3^; Fig. 4C). No significant correlation was found between the number of EPVS in the cerebrum and post-mortem interval to fixation (Pearson correlation coefficient = 0.060, *P* = 0.08) or post-mortem interval to imaging (Pearson correlation coefficient = −0.002, *P* = 0.94).

### Association of EPVS counts with age-related neuropathologies and vascular risk factors

Ordinal logistic regression combining neuropathologic, clinical, demographic and other factors as independent variables in the same model showed that the number of EPVS in the cerebrum was associated with microscopic infarcts (odds ratio (OR) = 1.47, 95% confidence interval (CI) = [1.12, 1.93], *P* = 0.0055) and diabetes mellitus (OR = 1.60, CI = [1.18, 2.18], *P* = 0.0027) after Bonferroni correction (Table 3). In the frontal lobe, the number of EPVS was associated with gross infarcts (OR = 1.57, CI = [1.19, 2.06], *P* = 0.0013). The numbers of EPVS in the temporal (OR = 1.23, CI = [1.06, 1.43], *P* = 0.0079) and occipital lobe (OR =1.24, CI = [1.07, 1.44], *P* = 0.0040) were associated with CAA severity. EPVS in the frontal (OR = 1.56, CI = [1.15, 2.11], *P* = 0.0042), parietal (OR = 1.56, CI = [1.15, 2.10], *P* = 0.0040) and temporal lobes (OR = 1.81, CI = [1.33, 2.47], *P* = 0.0002) were also associated with diabetes mellitus. The number of EPVS in the basal ganglia was associated with the history of hypertension (OR = 1.46, CI = [1.10, 1.94], *P* = 0.0081) and nearly significantly associated with microscopic infarcts (OR = 1.43, CI = [1.09, 1.86], *P* = 0.0090) (Table 3). A number of other associations did not survive Bonferroni correction (i.e. 0.0083 < *P* < 0.05) as can be seen in Table 3, most notably indicating additional regions where the number of EPVS may be associated with infarcts and diabetes, and suggesting that EPVS in the basal ganglia may also be associated with arteriolosclerosis. Repeating all analyses including one additional parameter for hippocampal sclerosis did not change the results. Finally, repeating all analyses including additional parameters to test for interactions with age showed no statistically significant influence of age on the above findings.

**Table 3:** Ordinal logistic regression on the association of the number of EPVS in the cerebrum, individual lobes and basal ganglia with each of the neuropathologies and clinical variables

	Cerebrum OR (95% CI)		Frontal lobe OR (95% CI)		Parietal lobe OR (95% CI)		Temporal lobe OR (95% CI)		Occipital lobe OR (95% CI)		Basal ganglia OR (95% CI)	
Gross infarcts	1.58 (1.23, 2.03)	1.40 (1.06, 1.84)	1.68 (1.31, 2.16)	**1.57** **(1.19, 2.06)**	1.31 (1.02, 1.68)	1.23 (0.94, 1.61)	1.27 (0.99, 1.64)	1.09 (0.82, 1.44)	1.28 (0.97, 1.68)	1.18 (0.88, 1.58)	1.64 (1.27, 2.10)	1.36 (1.03, 1.80)
Micro infarcts	1.50 (1.16, 1.92)	**1.47** **(1.12, 1.93)**	1.43 (1.11, 1.83)	1.34 (1.03, 1.76)	1.38 (1.08, 1.77)	1.39 (1.06, 1.81)	1.36 (1.05, 1.75)	1.34 (1.02, 1.77)	1.41 (1.07, 1.85)	1.34 (1.00, 1.79)	1.54 (1.20, 1.98)	1.43 (1.09, 1.86)
CAA	0.98 (0.86, 1.12)	-	0.99 (0.87, 1.13)	-	1.04 (0.91, 1.18)	-	1.11 (0.97, 1.27)	**1.23** (**1.06, 1.43)**	1.23 (1.06, 1.42)	**1.24** (**1.07, 1.44)**	0.87 (0.76, 0.99)	1.02 (0.88, 1.18)
Hypertension	1.19 (0.90, 1.55)	-	1.19 (0.91, 1.55)	-	1.08 (0.83, 1.41)	-	0.91 (0.69, 1.20)	-	1.10 (0.82, 1.49)	-	1.58 (1.21, 2.07)	**1.46** (**1.10, 1.94)**
Diabetes	1.72 (1.28, 2.31)	**1.60** **(1.18, 2.18)**	1.73 (1.29, 2.31)	**1.56** **(1.15, 2.11)**	1.58 (1.18, 2.12)	**1.56** **(1.15, 2.10)**	1.80 (1.34, 2.42)	**1.81** (**1.33, 2.47)**	1.30 (0.95, 1.79)	1.35 (0.98, 1.87)	1.72 (1.29, 2.31)	1.41 (1.03, 1.91)

This table has been shortened to show only rows with significant findings. The complete table can be found in the Supplementary material. The left column for each region shows the results of ordinal logistic regression with a single independent variable controlling for demographics, tissue volume, scanner, and post-mortem intervals to fixation and to imaging, and the right column shows the results of ordinal logistic regression including all variables that reached *P* < 0.20 in the first step. Significant findings in the latter ordinal logistic regression (*P* < 0.0083; Bonferroni correction) are shown in bold.

Gross Infarcts, presence of gross infarcts; Micro Infarcts, presence of microscopic infarcts; CAA, severity of cerebral amyloid angiopathy.

### Association of EPVS counts with level of cognition at death and rate of cognitive decline

Linear mixed-effects models showed that the number of EPVS in the cerebrum was associated with a lower level of global cognition [*β* = −0.044, standard error (SE) = 0.013, *P* = 0.0008] and lower level of four cognitive domains: episodic memory (*β* = −0.048, SE = 0.017, *P* = 0.004), semantic memory (*β* = −0.056, SE = 0.018, *P* = 0.0015), working memory (*β* = −0.037, SE = 0.012, *P* = 0.002) and visuospatial ability (*β* = −0.049, SE = 0.013, *P* = 0.0002) at the time of death, after Bonferroni correction, controlling for neuropathologies, clinical variables, demographics and hemisphere volume (Table 4). The number of EPVS was not associated with the level of perceptual speed or with the rate of decline in global cognition or any cognitive domain.

**Table 4:** Association of the number of EPVS in the cerebrum with the level of cognition at the time of death and rate of cognitive decline

	Association of EPVS number with level of cognition at time of death *β* (SE, *P*-value)	Association of EPVS number with cognitive decline *β* (SE, *P*-value)
Global cognition	**−0.044** (**0.013, 0.0008)**	−0.002 (0.001, 0.11)
Episodic memory	**−0.048** (**0.017, 0.004)**	−0.002 (0.001, 0.08)
Semantic memory	**−0.056** (**0.018, 0.0015)**	−0.003 (0.0015, 0.043)
Working memory	**−0.037** (**0.012, 0.002)**	−0.0009 (0.0011, 0.45)
Perceptual speed	−0.031 (0.014, 0.03)	−0.0004 (0.0012, 0.77)
Visuospatial ability	**−0.049** (**0.013, 0.0002)**	−0.002 (0.0011, 0.057)

Linear mixed-effects models included a composite cognitive score (for global cognition or each of the five cognitive domains) as the longitudinal dependent variable, the number of EPVS in the cerebrum as well as its interaction with the time before death as the independent variables, and were controlled for all the neuropathologies, clinical variables, demographics and hemisphere volume, as well as the interaction of each of these variables with the time before death. Significant findings (*P* < 0.0083; Bonferroni correction) are shown in bold.

## Discussion

The present work first developed a deep-learning model ensemble for automatic segmentation, localization and quantification of EPVS in *ex vivo* brain MRI data, and then used this new method to investigate the neuropathologic, clinical and cognitive correlates of EPVS in a large number of community-based older adults. Developing a new EPVS segmentation tool was necessary because, although *ex vivo* MRI offers a number of advantages over *in vivo* MRI, especially in studies combining MRI and pathology, none of the published EPVS segmentation tools can be used with the available *ex vivo* MRI data. The new deep-learning model ensemble exhibited high sensitivity in detecting EPVS as small as 3 mm^3^, good segmentation accuracy and high segmentation consistency. When applied to *ex vivo* MRI data from a large number of community-based older adults, the highest number of EPVS was observed in the frontal lobe, but the highest density of EPVS was observed in the basal ganglia. The total number of EPVS in the cerebrum as well as in the frontal lobe was independently associated with infarcts, while temporal and occipital lobe EPVS were associated with CAA severity. These findings suggest that EPVS share neurobiological pathways with infarcts and CAA and that these pathways are dependent on location within the brain. The total number of EPVS in the cerebrum as well as frontal, parietal and temporal EPVS were also associated with diabetes mellitus above and beyond the effects of neuropathologies, and basal ganglia EPVS were independently associated with hypertension, providing important new leads on the role of these vascular risk factors in the condition of the glymphatic system. Finally, EPVS were associated with lower levels of cognition above and beyond the effects of neuropathologies, clinical variables and demographics, suggesting that EPVS represent additional brain anomalies contributing to lower cognitive function.

Manual segmentation of EPVS is time-consuming and visual rating scales have important and well-known limitations such as detection bias and floor/ceiling effects. The new segmentation deep-learning model ensemble addressed the above limitations for the first time in an MRI and pathology investigation of EPVS. As a result, the present work extended previously published findings on the neuropathologic correlates of EPVS and enabled new discoveries mainly through the ability to localize EPVS.

The number of EPVS in the cerebrum and specifically in the frontal lobe was associated with microscopic and gross infarcts, respectively, while the numbers of EPVS in the temporal and occipital lobes were associated with CAA severity (after Bonferroni correction). The association with infarcts is in agreement with our previous work in community-based older adults as well as with *in vivo* studies linking EPVS to lacunar infarction and increased risk of stroke.^9-11,54-56^ However, the underlying mechanism is not well understood. Ischaemic insult in rodent brain has revealed that ischaemic spreading depolarizations and vasoconstriction lead to EPVS and increase the influx of CSF into brain tissue driving acute ischaemic swelling.^57^ Interestingly, multiple microscopic infarcts were sufficient to trigger widespread changes in the glymphatic system in a murine model.^58^ The association of EPVS with CAA is in agreement with investigations conducted in patients mainly suffering from CAA.^21^ The fact that this association was observed in the temporal and occipital lobes is of interest since the distribution of CAA generally peaks in posterior brain regions.^59^ Pathogenesis of CAA is thought to involve impaired perivascular drainage originating due to advanced age or other factors (e.g. vascular risk factors; see next paragraph), which traps Aβ in the perivascular space and increases Aβ aggregation and deposition along the basement membranes of vessels.^60^ In turn, as Aβ accumulates, it activates vascular injury pathways further impairing perivascular drainage and establishing a self-reinforcing loop.^61^ Although these pathways are complex and not fully understood, our finding on the association of EPVS with CAA is in support of this general concept. Overall, the present study bridges previously published work showing that EPVS are associated with infarcts^10^ with other work showing that EPVS are associated with CAA,^21,62^ provides robust evidence for both associations, and demonstrates for the first time that the limitations of EPVS visual rating scales and the location-dependent characteristics of EPVS were behind what previously appeared as a discrepancy across studies.

The total number of EPVS in the cerebrum, as well as frontal, parietal, and temporal EPVS, was associated with diabetes mellitus above and beyond the effects of neuropathologies, and basal ganglia EPVS were independently associated with hypertension (after Bonferroni correction). We and others have previously reported on the links of EPVS with these vascular risk factors in humans.^9,10,63-65^ Reduced glymphatic flow has also been reported in rat models of diabetes and hypertension.^66-68^ Here, the ability to quantify EPVS in different parts of the brain provided additional detail and demonstrated that the association of EPVS with diabetes is more widespread and present in almost all brain lobes, while the association with hypertension is more localized and present in the basal ganglia. In addition, the current study showed that these links are independent of age-related neuropathologies, suggesting independent pathways of diabetes and hypertension to EPVS formation. A recently proposed model suggests that accumulation of advanced glycation end products in diabetes and abnormal wall pulsatility in hypertension may be responsible for abnormal CSF flow patterns in perivascular spaces and reduced CSF influx into the parenchyma, leading to local inflammation, gliosis and atrophy of the parenchyma, stagnation of perivascular fluid and eventually dilation of the perivascular spaces.^69^ The above anomalies also lead to protein aggregation and may therefore partly contribute to CAA pathogenesis as described in the previous paragraph.

The number of EPVS in the cerebrum was associated with a lower level of global cognition, and more specifically a lower level of episodic memory, semantic memory, working memory and visuospatial ability, after Bonferroni correction, controlling for neuropathologies, clinical variables, demographics and hemisphere volume. These findings extend previous work in community cohorts that used visual rating scales and/or did not control for neuropathologies,^14,16,70^ and suggest that EPVS may represent additional brain tissue injury contributing to lower levels of cognition above and beyond the contributions of age-related neuropathologies and vascular risk factors. This additional brain tissue injury may be related to the local atrophy of the parenchyma around the perivascular spaces.

The present work has several important strengths and a few weaknesses. The strengths include (i) the large number of community-based older adults that participated in this study, (ii) the detailed neuropathologic examination that allowed controlling for the effects of comorbid pathologies in all analyses, (iii) imaging *ex vivo*, which reduces the bias towards lower frailty and ensures that imaging and neuropathologic examination capture the same brain condition, (iv) the development of a deep-learning model ensemble for automatic segmentation of EPVS specifically for *ex vivo* brain MRI, (v) thorough evaluation of the model ensemble also involving an expert neuroradiologist, (vi) full quantification of EPVS (not limited to a single slice) and (vii) localization of EPVS in the cerebrum. One weakness is the fact that laterality was not studied as only one cerebral hemisphere was imaged per participant. In addition, EPVS seen in *ex vivo* MRI were not confirmed *in vivo* in the same participants because such data were not available. However, it has already been established that there is good correspondence between EPVS seen *in vivo* and *ex vivo*,^17,71^ and therefore, the findings of the present work should be applicable to EPVS seen *in vivo*.

## Conclusion

A new deep-learning model ensemble for automated segmentation of EPVS was developed and used for full quantification and localization of EPVS in *ex vivo* brain MRI data from a large number of community-based older adults, making this the largest MRI and pathology study of EPVS conducted to date. We found strong evidence of an association between EPVS and vascular pathologies, specifically infarcts and CAA, and demonstrated that these links are dependent on location within the brain with the former association observed in the frontal lobe and the latter in the occipital and temporal lobes. Furthermore, associations of EPVS in most lobes with diabetes mellitus and of basal ganglia EPVS with hypertension independent of age-related neuropathologies support the notion of independent pathways from diabetes and hypertension to EPVS formation. Finally, EPVS were associated with a lower level of cognition above and beyond the effects of neuropathologies and vascular risk factors, suggesting that EPVS represent additional brain anomalies contributing to lower cognitive function. Overall, the present study used full quantification and localization of EPVS in a large number of community-based older adults and provided strong evidence on the vascular pathologies and vascular risk factors that are linked to EPVS, as well as indicated that tissue damage associated with EPVS has independent contributions to lower levels of cognition.

## Supplementary Material

fcae252_Supplementary_Data

## Data Availability

The data used in this work as well as the EPVS segmentation deep-learning model ensemble can be accessed by submitting a request to www.radc.rush.edu.
