# Supplementary material for: Quantitative assessment of enlarged perivascular spaces via deep-learning in community-based older adults reveals independent associations with vascular neuropathologies, vascular risk factors and cognition
Source: Brain Commun. 2024 Jul 30;6(4):fcae252. doi: 10.1093/braincomms/fcae252 (PMC11316207; doi:10.1093/braincomms/fcae252)
Supplement: fcae252_Supplementary_Data [file fcae252_supplementary_data.docx]

**Supplementary Material**

**Supplementary Table 1.** Ordinal logistic regression on the association of the number of EPVS in the cerebrum, individual lobes and basal ganglia with each of the neuropathologies and clinical variables. The left column for each region shows the results of ordinal logistic regression with a single independent variable controlling for demographics, tissue volume, scanner, and postmortem intervals to fixation and to imaging, and the right column shows the results of ordinal logistic regression including all variables that reached p<0.20 in the first step. Significant findings in the latter ordinal logistic regression (p<0.0083; Bonferroni correction) are shown in bold.

|  | **Cerebrum**  OR (95% C.I.) | | **Frontal lobe**  OR (95% C.I.) | | **Parietal lobe**  OR (95% C.I.) | | **Temporal lobe**  OR (95% C.I.) | | **Occipital lobe**  OR (95% C.I.) | | **Basal ganglia**  OR (95% C.I.) | |
| --- | --- | --- | --- | --- | --- | --- | --- | --- | --- | --- | --- | --- |
| Gross Infarcts | 1.58  (1.23, 2.03) | 1.40  (1.06, 1.84) | 1.68  (1.31, 2.16) | **1.57**  **(1.19, 2.06)** | 1.31  (1.02, 1.68) | 1.23  (0.94, 1.61) | 1.27  (0.99, 1.64) | 1.09  (0.82, 1.44) | 1.28  (0.97, 1.68) | 1.18  (0.88, 1.58) | 1.64  (1.27, 2.10) | 1.36  (1.03, 1.80) |
| Micro Infarcts | 1.50  (1.16, 1.92) | **1.47**  **(1.12, 1.93)** | 1.43  (1.11, 1.83) | 1.34  (1.03, 1.76) | 1.38  (1.08, 1.77) | 1.39  (1.06, 1.81) | 1.36  (1.05, 1.75) | 1.34  (1.02, 1.77) | 1.41  (1.07, 1.85) | 1.34  (1.00, 1.79) | 1.54  (1.20, 1.98) | 1.43  (1.09, 1.86) |
| CAA | 0.98  (0.86, 1.12) | - | 0.99  (0.87, 1.13) | - | 1.04  (0.91, 1.18) | - | 1.11  (0.97, 1.27) | **1.23**  **(1.06, 1.43)** | 1.23  (1.06, 1.42) | **1.24**  **(1.07, 1.44)** | 0.87  (0.76, 0.99) | 1.02  (0.88, 1.18) |
| Arteriolo  sclerosis | 1.13  (0.98, 1.31) | 1.10  (0.95, 1.29) | 1.12  (0.97, 1.29) | 1.07  (0.92, 1.25) | 1.05  (0.91, 1.21) | - | 1.12  (0.97, 1.30) | 1.11  (0.95, 1.29) | 1.05  (0.90, 1.23) | - | 1.26  (1.09, 1.45) | 1.20  (1.02, 1.40) |
| Athero  sclerosis | 1.07  (0.90, 1.26) | - | 1.11  (0.94, 1.30) | - | 0.97  (0.82, 1.14) | - | 0.91  (0.77, 1.07) | - | 1.02  (0.85, 1.23) | - | 1.18  (1.00, 1.39) | 1.06  (0.88, 1.27) |
| Aβ | 0.84  (0.75, 0.94) | 0.88  (0.77, 1.02) | 0.87  (0.78, 0.98) | 0.90  (0.78,1.03) | 0.85  (0.76, 0.95) | 0.89  (0.78, 1.02) | 0.89  (0.80, 1.00) | 0.90  (0.78, 1.04) | 1.02  (0.90, 1.15) | - | 0.80  (0.71, 0.89) | 0.89  (0.77, 1.02) |
| Tangles | 0.90  (0.83, 0.99) | 1.13  (1.00, 1.27) | 0.94  (0.86, 1.03) | 1.14  (1.01, 1.27) | 0.91  (0.83, 0.99) | 1.11  (0.99, 1.24) | 0.93  (0.85, 1.02) | 1.11  (0.98, 1.26) | 1.06  (0.96, 1.17) | - | 0.83  (0.76, 0.91) | 1.04  (0.93, 1.17) |
| Lewy bodies | 1.07  (0.82, 1.40) | - | 1.15  (0.88, 1.50) | - | 1.11  (0.85, 1.44) | - | 1.01  (0.77, 1.32) | - | 1.10  (0.82, 1.47) | - | 0.84  (0.65, 1.10) | - |
| LATE-NC | 0.88  (0.80, 0.97) | 0.93  (0.84, 1.04) | 0.86  (0.78, 0.95) | 0.90  (0.80, 1.00) | 0.91  (0.82, 1.00) | 0.94  (0.84, 1.04) | 0.93  (0.84, 1.03) | 0.99  (0.88, 1.10) | 0.95  (0.85, 1.06) | - | 0.85  (0.77, 0.94) | 0.96  (0.86, 1.07) |
| Heart disease | 0.79  (0.58, 1.07) | 0.71  (0.52,0.98) | 0.76  (0.56, 1.03) | 0.67  (0.49, 0.92) | 0.78  (0.58, 1.06) | 0.70  (0.51, 0.95) | 0.75  (0.55, 1.03) | 0.69  (0.50, 0.95) | 0.72  (0.51, 1.02) | 0.69  (0.49, 0.98) | 1.16  (0.86, 1.58) | - |
| Hypertension | 1.19  (0.90, 1.55) | - | 1.19  (0.91, 1.55) | - | 1.08  (0.83, 1.41) | - | 0.91  (0.69, 1.20) | - | 1.10  (0.82, 1.49) | - | 1.58  (1.21, 2.07) | **1.46**  **(1.10, 1.94)** |
| Diabetes | 1.72  (1.28, 2.31) | **1.60**  **(1.18, 2.18)** | 1.73  (1.29, 2.31) | **1.56**  **(1.15, 2.11)** | 1.58  (1.18, 2.12) | **1.56**  **(1.15, 2.10)** | 1.80  (1.34, 2.42) | **1.81**  **(1.33, 2.47)** | 1.30  (0.95, 1.79) | 1.35  (0.98, 1.87) | 1.72  (1.29, 2.31) | 1.41  (1.03, 1.91) |
| Smoking | 0.85  (0.67, 1.07) | 0.85  (0.67,1.08) | 0.85  (0.67, 1.07) | 0.84  (0.66, 1.06) | 0.83  (0.66, 1.05) | 0.83  (0.65, 1.05) | 0.77  (0.61, 0.97) | 0.79  (0.62, 1.01) | 0.78  (0.60, 1.01) | 0.78  (0.60, 1.02) | 0.90  (0.71, 1.14) | - |
| *APOE ε4* | 0.68  (0.51, 0.91) | 0.81  (0.59, 1.10) | 0.71  (0.54, 0.94) | 0.80  (0.59, 1.08) | 0.72  (0.54, 0.95) | 0.80  (0.59, 1.08) | 0.70  (0.53, 0.94) | 0.71  (0.52, 0.97) | 0.99  (0.72, 1.34) | - | 0.66  (0.50, 0.88) | 0.85  (0.62, 1.15) |

Abbreviations: Gross Infarcts, presence of gross infarcts; Micro Infarcts, presence of microscopic infarcts; CAA, severity of cerebral amyloid angiopathy; Arteriolosclerosis, severity of arteriolosclerosis; Atherosclerosis, severity of atherosclerosis; Aβ, composite measure of Aβ burden; Tangles, composite measure of tangle density; Lewy bodies, presence of Lewy bodies; LATE, LATE-NC stage; *APOE ε4*, presence of at least one copy of the *APOE ε4* allele.
